# Supplementary figures and images for: Linking prokaryotic genome size variation to metabolic potential and environment
Source: ISME Commun. 2023 Mar 27;3:25. doi: 10.1038/s43705-023-00231-x (PMC10042847; doi:10.1038/s43705-023-00231-x)

**Supplementary material 1**

Water column


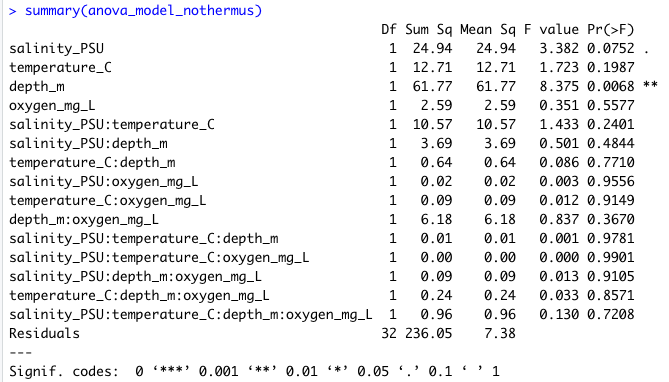


Sediments


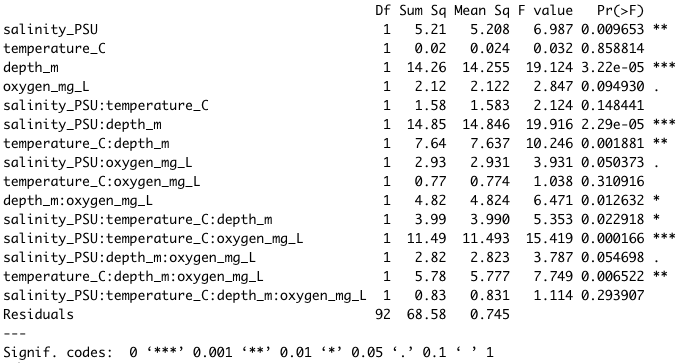

Supplement: Supplementary file 1 — Supplemental material 1 [file 43705_2023_231_MOESM1_ESM.docx]

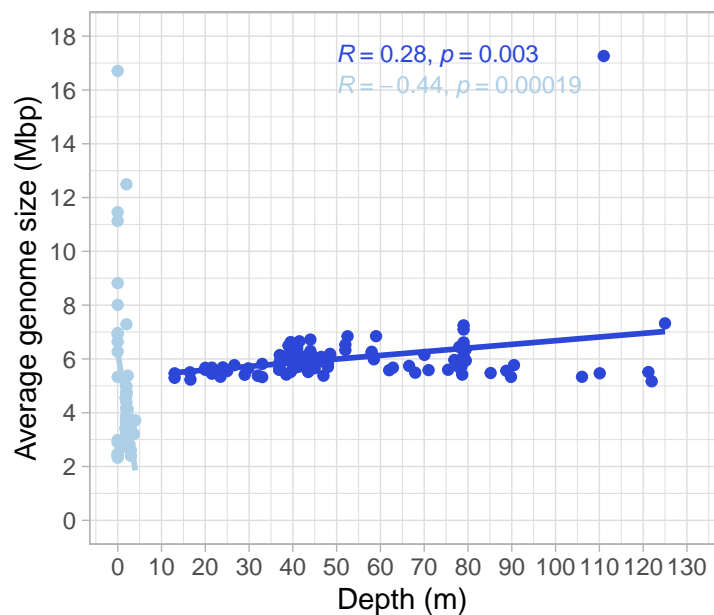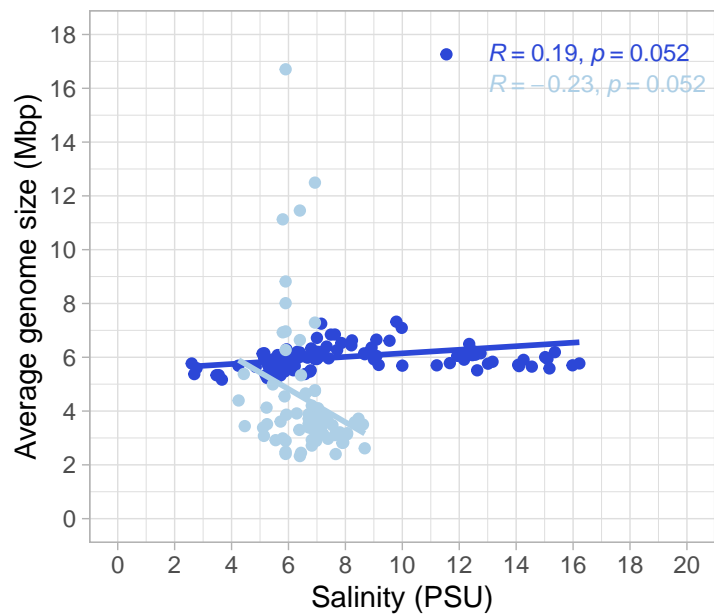

Supplement: Supplementary file 4 — Figure S1 [file 43705_2023_231_MOESM4_ESM.pdf]

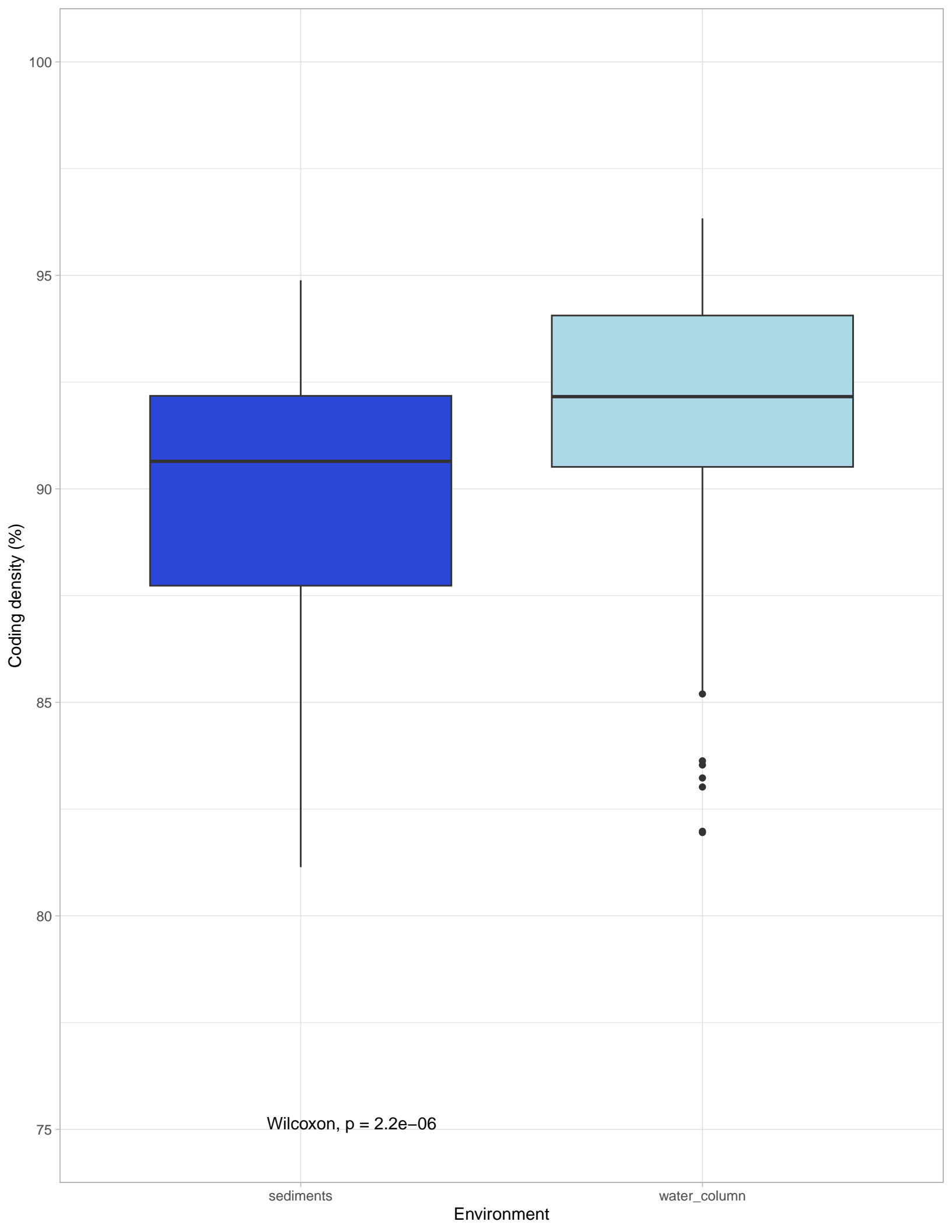

Supplement: Supplementary file 5 — Figure S2 [file 43705_2023_231_MOESM5_ESM.pdf]
