# Supplementary material for: Linking prokaryotic genome size variation to metabolic potential and environment
Source: ISME Commun. 2023 Mar 27;3:25. doi: 10.1038/s43705-023-00231-x (PMC10042847; doi:10.1038/s43705-023-00231-x)
Supplement: Supplementary file 2 — Supplemental material 2 [file 43705_2023_231_MOESM2_ESM.docx]

**Supplementary Material 3**

Completeness estimations in the figure below include all bins from stratfreshDB (Buck et al., 2021).

Completeness calculated with CheckM using markers determined by the default workflow in purple

Completeness calculated with CheckM using specific gene markers for Actinobacteria and Patescibacteria in orange.

* Denotes significant difference using Wilcoxon test


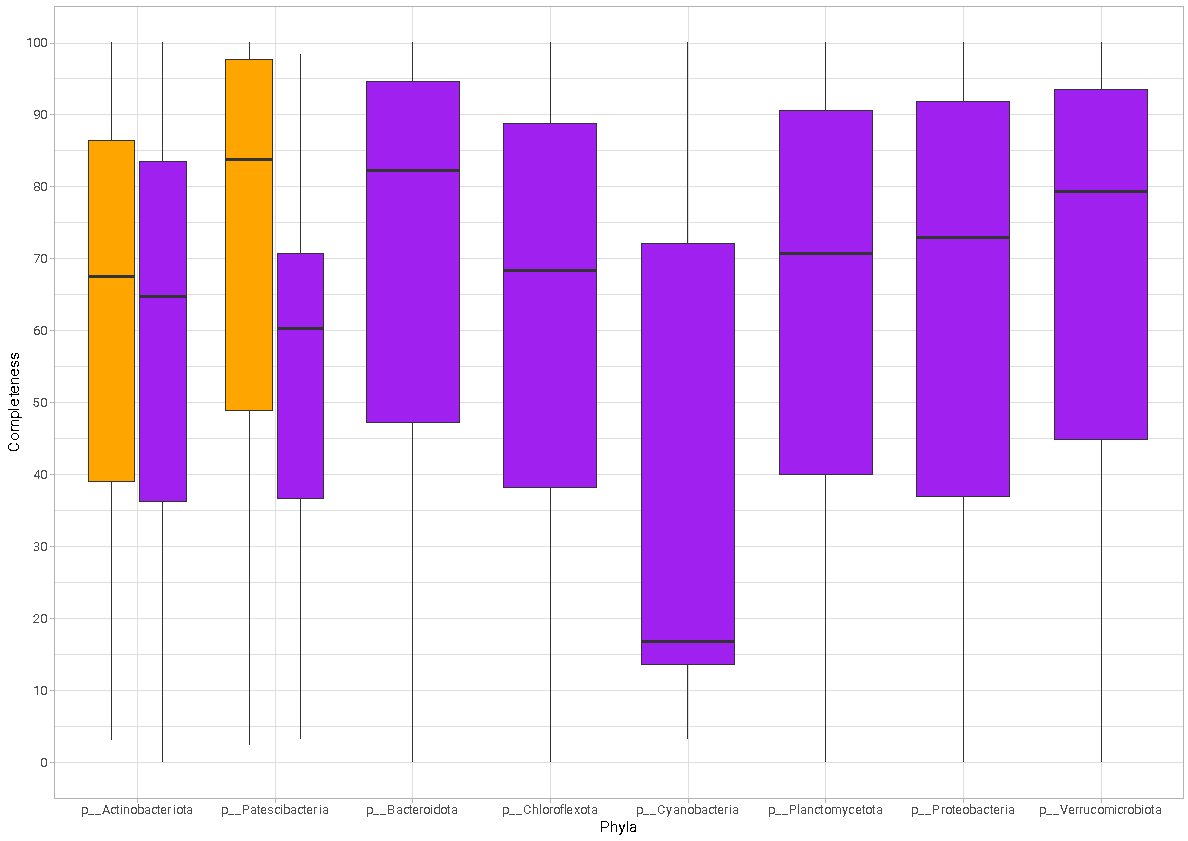


* *


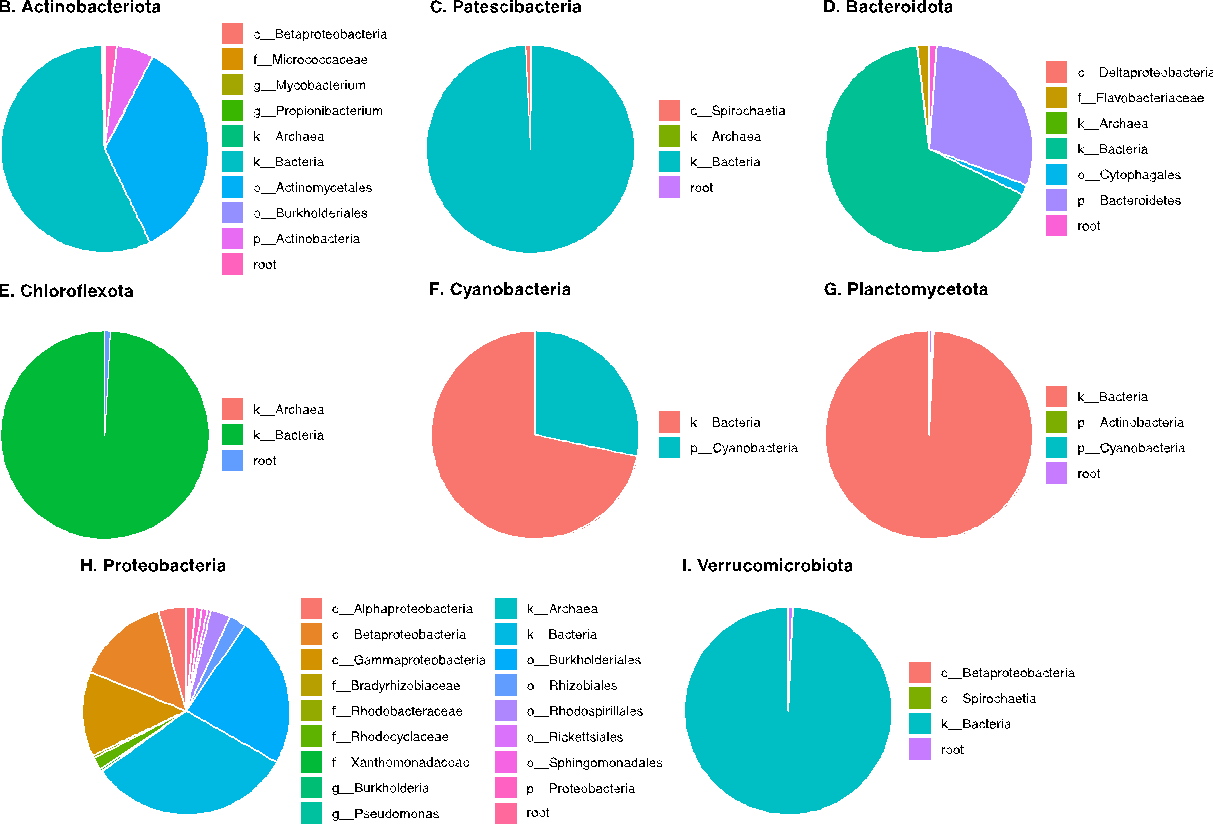


Lineage CheckM used to calculate completeness by default parameters.

References

Buck, M., Garcia, S. L., Fernández, L., Martin, G., Martinez-Rodriguez, G. A., Saarenheimo, J., et al. (2021). Comprehensive dataset of shotgun metagenomes from oxygen stratified freshwater lakes and ponds. Sci. Data 8:131. doi: 10.1038/s41597-021-00910-1.
